# The quality of working life questionnaire for cancer survivors (QWLQ-CS): factorial structure, internal consistency, construct validity and reproducibility

**DOI:** 10.1186/s12885-017-3966-1

**Published:** 2018-01-10

**Authors:** Merel de Jong, Sietske J. Tamminga, Robert J. J. van Es, Monique H. W. Frings-Dresen, Angela G. E. M. de Boer

**Affiliations:** 1 Coronel Institute of Occupational Health, Amsterdam Public Health research institute, Academic Medical Center, P.O. box 22660, 1100 DD Amsterdam, the Netherlands; 2 Department of Head and Neck Surgical Oncology, UMC Utrecht Cancer Center, University Medical Center Utrecht, P.O. box 85500, 3508 GA Utrecht, the Netherlands

**Keywords:** Quality of working life, Cancer survivors, Questionnaire, Return to work, Work continuation, Psychometric properties

## Abstract

**Background:**

To assess the factorial structure, internal consistency, construct validity and reproducibility of the Quality of Working Life Questionnaire for Cancer Survivors (QWLQ-CS).

**Methods:**

An Exploratory Factor Analysis (EFA) was performed on QWLQ-CS data from a sample of employed cancer survivors to establish the final number of items and factorial structure of the QWLQ-CS. Internal consistency was assessed using Cronbach’s alpha. In a second sample of (self-)employed cancer survivors, construct validity was tested by convergent validity (correlations of QWLQ-CS with construct-related questionnaires), and discriminative validity (difference in QWLQ-CS scores between cancer survivors and employed people without cancer). In a subgroup of stable cancer survivors subtracted from the second sample, reproducibility was evaluated by Intraclass Correlation Coefficient (ICC) and Standard Error of Measurement (SEM).

**Results:**

EFA on QWLQ-CS data of 302 cancer survivors resulted in 23 items and five factors. The internal consistency of the QWLQ-CS was Cronbach’s α = 0.91. Convergent validity on data of 130 cancer survivors resulted in *r* = 0.61–0.70. QWLQ-CS scores of these cancer survivors statistically differed (*p* = 0.04) from employed people without cancer (*N* = 45). Reproducibility of QWLQ-CS data from 87 cancer survivors demonstrated an ICC of 0.84 and a SEM of 9.59.

**Conclusions:**

The five-factor QWLQ-CS with 23 items and adequate internal consistency, construct validity, and reproducibility at group level can be used in clinical and occupational healthcare, and research settings.

**Electronic supplementary material:**

The online version of this article (10.1186/s12885-017-3966-1) contains supplementary material, which is available to authorized users.

## Background

By 2025, cancer incidence is expected to rise to 19.3 million cases worldwide [1]. As new treatments and screening instruments increase the chances of surviving cancer [2] and as more people work longer, an increasing number of cancer survivors are continuing to work or returning to employment [3]. Unfortunately, cancer survivors can encounter difficulties at work. Cancer survivors are 1.4 times more likely to be unemployed than ‘healthy’ employees [4] for example, and when cancer survivors are employed, they report facing psychological and physical difficulties at work [5, 6].

Although a cancer diagnosis can have a negative physical, cognitive and psychological impact on a person’s working life [7, 8], work also benefits cancer survivors. For instance, work allows them to maintain a sense of identity and self-esteem and provides financial security [9]. Getting adequate support from one’s general physician or the workplace is related to a successful return to work [10]. Yet, there are additional actors involved in the occupational rehabilitation of cancer survivors, such as occupational physicians, oncologists and other healthcare professionals [11, 12]. To provide adequate support to cancer survivors, these actors should be able to assess the overall work situation of the patient and not only work-related outcomes such as work productivity [13].

Cancer survivors perceive various difficulties in the workplace, such as coping with fatigue [14] or lack of understanding from their work environment [15]. These difficulties are likely to contribute to subjective work outcomes, such as Quality of Working Life (QWL). We define QWL as ‘the experiences and perceptions of cancer survivors in the work situation’ [16]. Previous research indicates that ‘healthy’ employees with a high QWL show lower levels of turnover intention [17, 18]. Research on QWL is often performed among ‘healthy’ employees. For instance, existing Quality of Working Life questionnaires [19–22] were developed for ‘healthy’ employees or particular occupations [23] and do not incorporate items on the effect of a cancer diagnosis and treatment, such as fatigue and anxiety.

To measure QWL among cancer survivors, and to take account of the impact of cancer diagnosis and treatment on a cancer survivors’ working life, we developed the self-administered Quality of Working Life Questionnaire for Cancer Survivors (QWLQ-CS) [24]. The development of the QWLQ-CS was based on the guidelines for developing Questionnaire Modules provided by the EORTC Quality of Life Group [25]. We generated items from the literature [26] and held focus groups with employed cancer survivors and interviews with oncological occupational physicians and employers [24]. An initial version was constructed and pre-tested among employed and self-employed cancer survivors which resulted in a preliminary version of the 104-item QWLQ-CS [16]. This article describes two field studies that were based on the last phase of questionnaire development [25]. The objective of field study I was to reduce the number of items in the preliminary QWLQ-CS and determine its factorial structure and internal consistency. The objective of field study II was to test the construct validity and reproducibility of the final version of the QWLQ-CS.

## FIELD STUDY I: Item reduction, factorial structure and internal consistency of the QWLQ-CS

### Methods

#### Design

Field study I was based on a cross-sectional design, with the aim of reducing the number of items in the preliminary QWLQ-CS and determining its underlying factorial structure. To guarantee a high level of methodological quality in evaluating the measurement properties of the instrument, the COSMIN checklist [27] was used. The Medical Ethics Committee of the Academic Medical Center (AMC) deemed ethical approval to be unnecessary (W14_323#14.17.0387).

#### Participants

Cancer survivors were recruited in Dutch hospitals (*N* = 3). The cooperating hospital departments were those of specialising in breast cancer, gastrointestinal cancer, dermatological oncology, gynaecological oncology, head and neck surgical oncology, oncological lung diseases, radiotherapy and urological oncology. After selection by patient administrations, cancer survivors were invited to participate by their oncological specialist during an appointment or by post. Cancer survivors were also recruited by issuing invitations through a Dutch online cancer platform and a patient organisation’s homepage. Furthermore, 12 cancer survivors who had been recruited for a previous study [16], but who had not participated because the required sample size had been achieved, received a new invitation.

Inclusion criteria were: (1) diagnosed with malignant cancer (2) diagnosed between three months and ten years ago, (3) currently between 18 and 65 years of age, (4) 18 years or older when diagnosed with cancer, (5) employed or self-employed, and participated in work in the last four weeks, and (6) fluent in Dutch. Exclusion criteria were: being diagnosed with a severe psychiatric disorder or receiving palliative treatment. The recruitment strategy via the Dutch hospitals and Dutch online cancer platform allowed only for a pre-selection on a few inclusion criteria (e.g., age, diagnosis) as no more demographics were available. Therefore, the other inclusion criteria were checked upon response by a participant prior to participation.

### Informed consent

If cancer survivors wanted information about the study or wished to participate, they consented to being contacted by the research team. Next, all cancer survivors who agreed to participate by telephone received an informed consent form for study participation by post, which had to be signed.

### Procedure

Data collection took place between May 2015 and December 2015. Cancer survivors were asked to complete the preliminary QWLQ-CS in paper or digital form. The digital version of the QWLQ-CS was designed using the online survey software Fluidsurveys (SurveyMonkey Europe, Ireland 2014).

### Instruments

#### Preliminary version of the QWLQ-CS

The preliminary QWLQ-CS was developed in Dutch and consisted of 104 items. Positively and negatively phrased items could be answered on a 6-point Likert scale without numbers (Totally disagree - Totally agree). The items had a reference period of the past four weeks. The extra response option ‘Not applicable’ was provided for cases in which cancer survivors felt an item was not applicable to their work or health situation (e.g., if self-employed cancer survivors were asked to answer items about their immediate supervisor or colleagues).

#### Other variables

Demographic, health- and work-related variables were assessed (Table 1).

**Table 1:** Sample characteristics field study I and II

		Field study I		Field study II					
Sample population		Cancer survivors		Cancer survivors^a^		Cancer survivors^b^		Healthy Employees^c^	
Sample size		*N* = 302		*N* = 130		*N* = 87		*N* = 45	
*Demographic characteristics*									
Age (mean in years ± standard deviation)		52 ± 8		52 ± 8		52 ± 9		51 ± 9	
		*N*	(%)	N	(%)	N	(%)	N	(%)
Gender - male		83	(28)	26	(20)	17	(20)	9	(20)
Marital status	Married/living together with a partner	240	(79)	106	(82)	69	(79)	38	(82)
Ethnical background	Dutch	279	(92)	123	(95)	82	(94)	43	(96)
	Immigrant first and second generation	21	(7)	7	(5)	5	(6)	2	(4)
*Clinical characteristics*									
Number of cancer diagnoses	1 diagnosis	256	(85)	109	(84)	75	(86)	–	–
	≥ 2 diagnoses	45	(15)	21	(16)	12	(14)	–	–
Cancer diagnosis^d^	Breast cancer	123	(36)	68	(49)	51	(55)	–	–
	Gynecological cancer	59	(17)	20	(14)	10	(11)	–	–
	Gastrointestinal cancer	47	(14)	34	(24)	22	(24)	–	–
	Urological cancer	36	(11)	0	(0)	0	(0)	–	–
	Hematological cancer	26	(8)	4	(3)	3	(3)	–	–
	Head and neck cancer	22	(6)	6	(4)	3	(3)	–	–
	Malignant melanomas	10	(3)	5	(4)	2	(2)	–	–
	Others (e.g. metastases)	17	(5)	3	(2)	2	(2)	–	–
Most recent cancer diagnosis	< 1 year ago	60	(20)	21	(16)	13	(15)	–	–
	1–3 years ago	162	(54)	63	(49)	41	(47)	–	–
	4–6 years ago	55	(18)	43	(33)	30	(35)	–	–
	> 6 years ago	24	(8)	3	(2)	3	(3)	–	–
Current cancer treatment	Yes	42	(14)	26	(20)	16	(18)	–	–
Cancer treatment^d^	Surgery	253	(39)	112	(39)	74	(37)	–	–
	Radiotherapy	152	(23)	60	(21)	45	(22)	–	–
	Chemotherapy	150	(23)	74	(26)	53	(26)	–	–
	Hormone therapy	67	(10)	34	(12)	23	(11)	–	–
	Other^e^	31	(5)	8	(3)	8	(4)	–	–
Co-morbidity	Yes	76	(25)	39	(30)	23	(26)	–	–
*Work characteristics*									
Education	Primary/secondary education	55	(18)	24	(18)	18	(21)	5	(11)
	Intermediate vocational education	102	(34)	51	(39)	36	(41)	15	(33)
	Higher prof/academic education	143	(47)	54	(42)	33	(38)	25	(56)
Work contract	Permanent position	225	(75)	91	(70)	64	(74)	35	(78)
	Temporary employment	19	(6)	12	(9)	7	(8)	1	(2)
	Self-employed	44	(15)	23	(18)	13	(15)	8	(18)
Contract hours	<12 h	12	(4)	3	(2)	1	(1)	1	(2)
	12–36 h	141	(47)	80	(62)	57	(66)	32	(71)
	>36 h	112	(37)	32	(25)	19	(22)	9	(20)
Current work hours	Total contract hours	193	(64)	94	(72)	62	(71)	–	–
	Proportion of contract hours (1–36)	108	(36)	36	(28)	25	(29)	–	–
Years on the job	0–3 years	36	(12)	20	(15)	12	(14)	7	(16)
	4–7 years	40	(13)	12	(9)	8	(9)	5	(11)
	> 8 years	225	(74)	67	(77)	98	(75)	33	(74)
Management position	Yes	78	(26)	25	(19)	17	(20)	8	(18)
Occupational sector	Health care and pharmacy	73	(24)	38	(29)	26	(30)	12	(27)
	Educational	34	(11)	8	(6)	5	(6)	8	(18)
	Government	30	(10)	14	(11)	11	(13)	4	(9)
	Industrial/production	20	(7)	8	(6)	5	(6)	2	(4)
	Facility management	12	(4)	4	(3)	2	(2)	1	(2)
	Wholesale/retail business	15	(5)	9	(7)	7	(8)	3	(7)
	Transport/logistics	16	(5)	6	(5)	4	(5)	1	(2)
	Business services	26	(9)	16	(12)	8	(9)	6	(13)
	Juridical	11	(4)	2	(2)	1	(1)	2	(4)
	IT	7	(2)	4	(3)	3	(3)	1	(2)
	Other	57	(19)	21	(16)	15	(17)	5	(11)
Monthly income	≤ €1000	46	(15)	21	(16)	13	(15)	6	(13)
	€1001 - €3000	125	(41)	85	(65)	60	(69)	25	(56)
	≥ €3001	98	(33)	12	(9)	8	(9)	11	(24)
Breadwinner position	Sole or shared	251	(83)	99	(76)	68	(78)	35	(78)

^a^Sample of cancer survivors at baseline

^b^Stable subgroup of cancer survivors who indicated no change in their health/work situation within the last four weeks

^c^Employed people without cancer or other physical/mental limitations affecting their job performance

^d^Percentages equal total diagnoses/treatments

^e^e.g. stem cell transplant, immunotherapy, bladder irrigation, no active treatment, alternative treatment

### Data analysis

The answers on the digital QWLQ-CS were directly exported from the online survey software Fluidsurveys to the software IBM SPSS Statistics 23. The researchers entered the paper versions of the QWLQ-CS into Fluidsurveys twice. The data entry for two of every ten (20%) paper versions of the QWLQ-CS was checked by exporting the data to SPSS and calculating the margin of error. If ≥2% of the data entry was wrong, all of the paper versions were checked by a different researcher.

### Explorative factor analysis

An important first step in testing a new questionnaire is to assess its content by determining if the variables of the construct to be measures are related. Therefore it is necessary to assess the underlying factor structure of this new set of variables with an EFA. The EFA was performed on the 104-item QWLQ-CS in seven steps (Table 2). In step 1, each item was removed if it fulfilled one of two conditions. The first condition was aimed at preventing an uneven distribution of answers, which might lead to an inability to detect any improvement or to distinguish between patients [28]. The second condition was aimed at removing non-generic items. For instance, items were removed if ≥20% of cancer survivors had answered ‘Not applicable’. Step 2 assessed the inter-item correlation matrix. Items were removed if they had extremely low correlations (<0.2) with ≥80% of the other items, on the grounds that they were not related to any of the other items and were not measuring the same construct, or if they correlated too highly (>0.9) with other items, which implied that the content of these items was too similar [29].

**Table 2:** Steps in Exploratory Factor Analysis (EFA)

Input:		104-items preliminary QWLQ-CS
	Aim	Outcome/conditions
Step 1	Item deletion	• If ≥95% of the responses on an item was located in one response category • If ≥20% of the responses on an item was located in the ‘not applicable’ category AND this was specific to a subgroup
Step 2	Item deletion	• If an item correlated ≤0.2 with ≥80% of the other items • If two items correlated ≥0.9
Step 3	Test assumptions PCA	• Adequate sample size if Kaiser-Meyer-Olkin value >0.6 • Items were correlated if Bartlett’s test of sphericity *p* < 0.05
Step 4	Explore number of factors	• Outcome on Catell’s scree test • Outcome on Parallel Analysis
Step 5	Determine rotation for factor structure	• Outcome rotation (e.g. varimax, Quartimax, Direct Oblimin)
Step 6	Determine number of factors and items	• Analyzed per outcome of step 4: the number of items, item content, and item factor loadings • Assigned to a factor: items with factor loading >0.5
	Item deletion	• If item had a factor loading of <0.5 • If item had factor loadings of >0.3 on more factors: deletion discussed based on importance of item
Step 7	Item deletion	• If inter-item correlation ≥0.7 • If item had low inter-item correlation (0.2–0.4) with half of the items in the factor • If Cronbach’s alpha <0.7
Output:		Final QWLQ-CS

In order to perform the Principal Component Analysis (PCA) in IBM SPSS Statistics 23, the test assumption had to be met in step 3. The Kaiser-Meyer-Olkin test was used to assess the sample adequacy, and if this value was >0.6, the sample size was sufficient. For items to be correlated, Bartlett’s test of sphericity had to be *p* < 0.05 [30]. In step 4, the number of underlying factors in the QWLQ-CS was explored by analysing the outcomes on Catell’s scree test [31] and Parallel Analysis (PA). In a scree test, the number of factors are based on the break in the plot [32]. PA was used to compare the outcomes of the PCA eigenvalue of our data set to the mean eigenvalue of 100 random data set with the same number of items and sample size [33]. To determine the best fit for the rotation structure in step 5, the PCA was performed on a fixed set of factors, resulting from the scree test and PA and with various rotation methods (Table 2). Based on the rotation plots, we decided which rotation best fitted the data. In step 6, the final decision on the number of factors was made by carefully examining the number of items, their content and the items’ factor loadings on the different number of factors that had been retrieved in step 4. Items with a factor loading of >0.5 were allocated to that factor [29]. Items with a factor loading of <0.5 were removed, and a new PCA was performed after the removal of each item in order to analyse the new factor loadings of the items. For items with factor loadings of >0.3 on more than one factor, removal was discussed, because the interpretation of this item might be ambiguous [29].

Finally, in step 7 items were removed by analysing the internal consistency per factor. The internal consistency indicates the interrelatedness of the scale of the extent to which items assess the same construct [29]. Multiple parameters of internal consistency were analysed (Additional file 1). An item was deleted if it had an inter-item correlation of ≥0.7 with another item, and if it had low inter-item correlations (0.2–0.4) with half of the items in that factor. Finally, a Cronbach’s alpha between 0.7 and 0.9 was acceptable [29], with >0.9 suggesting a high level of item redundancy [28]. Therefore, items were deleted when the Cronbach’s alpha of the factor was <0.7 and >0.9. One PCA was performed to examine the stability of the factor structure.

## Results

Of the 1617 cancer survivors who were pre-selected on a selection of inclusion criteria (e.g., on age, diagnosis) and invited, a total of 490 cancer survivors responded. Of this group 308 cancer survivors met the other inclusion criteria as well and agreed to participate, and 182 cancer survivors did not met the other inclusion criteria (e.g., not employed) or responded to indicate they were not interested in participation. Ultimately, 302 cancer survivors completed the QWLQ-CS (Table 1).

### Explorative factor analysis (EFA)

In step 1, there were no items for which ≥95% of the responses fell into one category. However, 14 of the 104 items were removed because ≥20% of cancer survivors indicated that this item was not applicable to them (Table 2). None of the items in step 2 correlated ≥0.9 with other items, but four items did correlate ≤0.2 with ≥80% of the other items and were removed. In step 3, the PCA was therefore performed with 86 items. Test assumptions were achieved; the Kaiser-Meyer-Olkin test was 0.86 and Bartlett’s test of sphericity was significant (*p* < .001). In step 4, Catell’s scree test yielded four factors and PA identified eight factors. Varimax rotation was the best fit for the data in step 5. After carefully examining the number of factors resulting from the scree test and PA, the number of items, their content and the items factor loadings, a five-factor structure was determined in step 6. We removed 21 items because they had a factor loading below 0.5, and two items that showed overly high (above 0.3) loadings on other than their main factor. In step 7, 40 of the 63 remaining items were deleted based on inter-item correlations of ≥0.7, inter-item correlation between 0.2–0.4 of multiple items, or the scale’s Cronbach’s alpha of <0.7. This resulted in a total of 23 items that were divided into the subscales: 1) Meaning of work, 2) Perception of the work situation, 3) Atmosphere in the work environment, 4) Understanding and recognition in the organisation, and 5) Problems due to the health situation. These five factors explained 51% of the variance and the QWLQ-CS had good internal consistency (Cronbach’s alpha = 0.91). The Cronbach’s alpha of the subscales varied between 0.83 and 0.86 (Table 3).

**Table 3:** Exploratory Factor Analysis (EFA): factor loadings on five-factor structure

Item No.^a^	Items	Factors^b^				
		1	2	3	4	5
Subscale 1: Meaning of work (Cronbach’s α = 0.83)						
1.	Working gives me structure in my life	**0.87**	0.17	0.06	0.10	0.08
2.	I think it is good to work	**0.83**	0.04	0.12	0.13	−0.01
3.	I consider that my work gives me a goal in life	**0.69**	0.16	0.27	0.19	0.05
4.	I consider my work important	**0.67**	0.30	0.27	0.10	0.11
Subscale 2: Perception of the work situation (α = 0.85)						
5.	I do my work well	0.13	**0.82**	0.09	0.07	0.05
6.	I am self-confident in my work	0.12	**0.81**	0.13	0.08	0.19
7.	I am suited to my work	0.22	**0.80**	0.19	0.09	0.08
8.	I have control over the work I do	0.10	**0.72**	0.27	0.17	0.18
9.	I feel powerless in my work^c^	0.07	**0.52**	0.39	0.27	0.30
Subscale 3: Atmosphere in the work environment (α = 0.86)						
11.	I have the feeling I am taken seriously by people in my working environment	0.07	0.20	**0.78**	0.34	0.09
13.	I have good relations with my colleagues	0.16	0.09	**0.76**	0.17	−0.04
10.	I feel there is a positive atmosphere in my working environment	0.21	0.18	**0.73**	0.22	0.07
14.	I feel valuable to my colleagues	0.20	0.26	**0.71**	0.13	0.22
12.	I am content with my work	0.22	0.40	**0.55**	0.23	0.16
Subscale 4: Understanding and recognition in the organization (α = 0.85)						
18.	I am content with the fringe benefits provided by my employer	0.10	0.18	0.12	**0.78**	0.01
15.	My immediate superior understands my health situation and possible health problems	0.21	0.02	0.32	**0.75**	0.01
17.	I consider that employees with health problems are treated well in my organization	0.01	0.07	0.22	**0.74**	0.14
19.	I am content with my current income	0.12	0.13	0.04	**0.67**	0.14
16.	I have good relations with my immediate superior	0.15	0.09	0.35	**0.63**	−0.01
Subscale 5: Problems due to the health situation (α = 0.84)						
20.	Because of my health situation I have problems in my work with fatigue and/or lack of energy^c^	0.02	0.03	0.05	0.13	**0.84**
21.	I am limited in my work by my health situation^c^	0.04	0.16	0.02	0.07	**0.81**
22.	Because of my health situation I have little trust in my own body^c^	0.08	0.11	0.07	−0.05	**0.78**
23.	Because of my health situation I feel uncertain about the future^c^	0.04	0.19	0.13	0.10	**0.78**

^a^The item numbers correspond with the order in the QWLQ-CS

^b^Highest factor loading in bold

^c^These items have reversed scoring

## FIELD STUDY II: Construct validity and reproducibility of the QWLQ-CS

### Methods

#### Design

In field study II, we evaluated the psychometric properties of the final version of the QWLQ-CS. The study used two measurements: at baseline and at follow-up after four weeks. The measurement at baseline was executed to test the construct validity (i.e. convergent validity and discriminative validity) of the QWLQ-CS. The measurements at baseline and the four-week follow-up were used to determine its reproducibility. Again, we used the COSMIN checklist, and ethical approval from the Medical Ethics Committee of the AMC was deemed unnecessary (W14_323#14.17.0387).

### Participants

#### Cancer survivors

The recruitment process and inclusion criteria were similar to those used in field study I. Cancer survivors who had signed up for participation in field study I, but who had not participated because the sample size was sufficient, were included in field study II. Furthermore, the sample was completed by cancer survivors who were recruited from the patient administrations of the departments of breast cancer, gastrointestinal cancer and haematological cancer in three different hospitals.

#### Employed people without cancer

To assess the discriminative validity of the QWLQ-CS, a sample of employed people without cancer or other physical/mental limitations affecting their job performance was recruited. An item in the questionnaire verified whether the participant met these criteria. The participants were recruited by asking participating cancer survivors to voluntarily pass on information about the study and the participation form to an employed friend, relative, neighbour or colleague of the same sex and age. Furthermore, recruitment was undertaken within the hospital (e.g., via the website and research boards). It was not necessary to gain informed consent because the participants participated voluntary and the questionnaire was anonymous.

### Procedure

Data were collected between March 2016 and April 2016. At baseline, cancer survivors were asked to complete the questionnaire that comprised the QWLQ-CS, the Copenhagen Psychosocial Questionnaire subscales (COPSOQ) [34], the return-to-work self-efficacy scale (RTW-SE) [35], the 36-Item Short Form Health Survey subscale (SF-36) [36, 37], three items measured on a Visual Analogue Scale (VAS), demographic items, and health- and work-related items. At the four-week follow-up, cancer survivors were asked again to fill in the QWLQ-CS and two anchor questions. Employed people without cancer or other physical/mental limitations affecting their job performance also completed a paper or digital questionnaire comprising the QWLQ-CS, demographic items and work-related items.

### Instruments

#### Final version of the QWLQ-CS

The final version of the QWLQ-CS consisted of 23 items (Additional file 2). The overall QWLQ-CS score and sum scores of the subscales are calculated with a standardised score of 0–100, and at least 50% of the items need to be answered. Scores on negative items were reversed (*N* = 5). The scores are calculated by: ((sum of item scores – lowest possible score)/ range between lowest and highest possible score)*100. A higher score corresponds with a better QWL. Responses were given on a 6-point Likert scale (Totally disagree - Totally agree). The extra response category ‘Not applicable’ was available for items related to the work situation of self-employed cancer survivors, such as items about colleagues or supervisors. These items were analysed as missing.

#### *Copenhagen Psychosocial Questionnaire (COPSOQ)* subscales

Included were three COPSOQ subscales: ‘Meaning of work’, ‘Social community at work’, and ‘Social support from supervisors’ [34]. All subscales are scored 0–100 points, with a higher score indicating higher value on the subscale. The COPSOQ is a valid and reliable questionnaire for employees [34].

#### Return-to-work self-efficacy scale (RTW-SE)

The RTW-SE is an 11-item scale that consist of statements about the participant’s job. The scale score was calculated by computing a mean score. A higher score indicates better self-efficacy. The RTW-SE is validated among people with mental health problems [35].

#### 36-item short form health survey (SF-36) subscale

Included was the subscale ‘Role limitations due to physical health problems’ (score range 0–100). A higher score corresponded with less role limitations. The SF-36 has been validated in a population with cancer [38, 39].

#### Visual analogue scale (VAS)

Three items with a VAS measured overall QWL, overall work satisfaction and satisfaction with fringe benefits. The scores on all items ranged from 0 to 100, with a higher score referring to a higher QWL or level of satisfaction. The VAS is a valid and reliable instrument for measuring quality of life [40] and is widely used in cancer research [41].

#### Other variables

The same demographic, health- and work-related variables as in field study I were assessed. Furthermore, to assess the reproducibility of the QWLQ-CS, we identified a stable subgroup of cancer survivors who responded to the following two anchor questions with ‘no’: ‘Did a major change take place in your health situation/work situation within the last four weeks?’

### Data analysis

#### Construct validity

To measure the construct validity of the QWLQ-CS, convergent and discriminative validity were analysed at baseline (Additional file 1).

#### Convergent validity

Convergent validity of the QWLQ-CS was assessed by calculating the correlation between the QWLQ-CS, or one of its subscales, and existing reliable and valid scales or questionnaires that measure similar constructs. It was expected that the scales would correlate, and eight hypotheses about the magnitude and direction of the Spearman’s correlation coefficient were formulated (Table 4). Convergent validity was considered sufficient if ≥75% of the hypotheses were confirmed [29].

**Table 4:** Convergent validity: Spearman’s correlation coefficients

QWLQ-CS	Comparable construct	Hypothesis	Spearman’s correlation coefficient^a^
Total score	VAS overall QWL	*r* ≤ 0.7	**0.70**
	VAS overall work satisfaction	*r* ≤ 0.7	**0.61**
Subscale 1	COPSOQ meaning of work	*r* = 0.4–0.6	0.34
Subscale 2	RTW-SE	*r* = 0.4–0.6	**0.53**
Subscale 3	COPSOQ social community	*r* = 0.4–0.6	**0.58**
Subscale 4	COPSOQ support supervisors	*r* = 0.4–0.7	**0.61**
	VAS satisfaction fringe benefits	*r* = 0.4–0.6	**0.53**
Subscale 5	SF-36 role limitations	*r* = 0.4–0.7	**0.63**

^a^Confirmed hypotheses in bold

#### Discriminative validity

Hypotheses about expected differences in QWL between two groups indicated discriminative validity of the QWLQ-CS [29]. The scores of cancer survivors on the QWLQ-CS were compared to those of employed people without cancer or other physical/mental limitations affecting their job performance. The response option ‘Not applicable’ was available for subscale 5 ‘Problems due to the health situation’. It was hypothesised that the outcomes on the QWLQ-CS would differ, with cancer survivors getting lower QWLQ-CS scores. The Mann-Whitney U-test assessed whether there were statistical significant differences between the two groups (*p* ≤ 0.05) [30].

#### Reproducibility

The reproducibility of the QWLQ-CS was assessed by test-retest reliability and the level of agreement (Additional file 1), measured at baseline and the four-week follow-up in the stable subgroup of cancer survivors. The Intraclass Correlation Coefficient with absolute agreement (ICC_agreement_) for the QWLQ-CS and subscales was calculated as a measure of test-retest reliability. We accepted an ICC_agreement_ of ≥0.70 for use at group level [42]. Next, we measured the level of agreement by calculating the Standard Error of Measurement with absolute agreement (SEM_agreement_) of the QWLQ-CS and its subscales [43]. To detect any statistical errors between the measurements at baseline and the four-week follow-up, a t-test was performed to analyse whether the mean differences differed from zero in a statistically significant way [44]. Finally, we analysed the Limits of Agreement (LoA) by constructing a Bland and Altman plot [45]. In addition, a 95% Confidence Interval (CI) was calculated to assess the variability in the estimated limits [46].

#### Floor and ceiling effects

When >15% of the cancer survivors scored the lowest or highest possible score on the QWLQ-CS or its subscales, this was considered an indication of a floor or ceiling effect [29].

## Results

### Construct validity

The sample at baseline consisted of 130 cancer survivors (Table 1). The score average and standard deviations on the QWLQ-CS and its subscales are displayed in Table 5.

**Table 5:** Intraclass Correlation Coeffient (ICC) and Standard Error of Measurement (SEM) of the stable subgroup of cancer survivors

QWLQ-CS	Total sample^1^						Stable subgroup^2^											
	Baseline			4 weeks follow-up			Baseline			4 weeks follow-up			Difference baseline – 4 weeks follow-up		SEM	ICC*	ICC 95% CI	
	N	Mean	SD	N	Mean	SD	N	Mean	SD	N	Mean	SD	Mean	SD			Lower bound	Upper bound
Subscale 1	130	80.35	13.51	100	81.65	12.63	87	80.52	12.23	86	81.45	12.95	0.87	9.74	12.57	**0.70**	0.58	0.80
Subscale 2	130	81.48	10.49	101	80.32	10.73	87	81.89	9.21	87	80.78	10.93	−1.10	9.40	10.11	0.57	0.41	0.69
Subscale 3	129	81.10	10.92	101	81.13	10.52	86	81.25	11.44	87	82.04	10.25	0.97	8.68	10.87	0.68	0.55	0.78
Subscale 4	112	74.82	13.97	88	75.55	14.15	77	75.55	14.26	76	75.95	13.39	0.38	7.55	13.89	**0.85**	0.77	0.90
Subscale 5	130	56.89	24.32	101	56.09	24.61	87	57.70	24.11	87	58.22	24.24	0.52	11.96	24.17	**0.88**	0.82	0.92
Total score	130	75.47	9.82	101	75.39	9.75	87	75.94	9.70	87	76.17	9.50	0.23	5.44	9.59	**0.84**	0.77	0.89

^1^Sample of cancer survivors in field study II

^2^Stable subgroup of cancer survivors in field study II who indicated no change in their health/work situation within the last four weeks

*Confirmed hypotheses in bold

#### Convergent validity

Spearman’s correlation coefficients between the overall QWLQ-CS score and VAS overall quality of working life was 0.70, and with VAS overall work satisfaction 0.61 (Table 4). The correlation between QWLQ-CS subscale 1 ‘Meaning of work’ and COPSOQ subscale ‘Meaning of work’ was 0.34, and between QWLQ-CS subscale 2 ‘Perceptions of the work situation’ and RTW-SE 0.53. A correlation of 0.58 was found for QWLQ-CS subscale 3 ‘Atmosphere in the work environment’ and COPSOQ subscale ‘Social community at work’. Correlations of 0.61 and 0.53 were found for QWLQ-CS subscale 4 ‘Understanding and recognition in the organisation’ and COPSOQ subscale ‘Support from supervisors’ and VAS satisfaction with fringe benefits respectively. The correlation between QWLQ-CS subscale 5 ‘Problems due to the health situation’ and SF-36 subscale ‘Role limitations’ was 0.63. Overall, ≥75% of the a priori hypotheses were confirmed.

#### Discriminative validity

Employed people without cancer or other physical/mental limitations affecting their job performance (*N* = 45) completed the QWLQ-CS (Table 1). Statistically significant differences were found between their overall QWLQ-CS mean score (*M* = 79) with a Standard deviation (*SD* = 11) and that of cancer survivors (*M* = 75, *SD* = 10) (*p* = 0.04). There were significant statistical differences between the mean scores on subscale 5 ‘Problems due to the health situation’ (*p* = 0.00) for employed people without cancer (*M* = 81, *SD* = 16) and cancer survivors (*M* = 57, *SD* = 24). There were no statistically significant differences (*p* = 0.13–0.95) on other subscales.

### Reproducibility

Of the sample at baseline (*N* = 130), 100 cancer survivors completed the questionnaire at follow-up (23% lost to follow-up). Eighty-seven cancer survivors who indicated no change in response to the two anchor questions were allocated to the stable subgroup and their QWLQ-CS and subscales scores are displayed in Table 5.

**Fig. 1:**
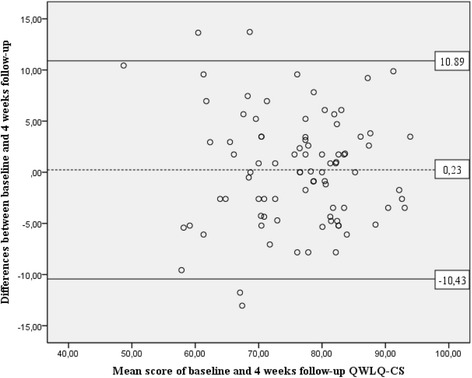
Bland and Altman plot for QWLQ-CS of the stable subgroup of cancer survivors (*N* = 87)

The single measures ICC_agreement_ for the overall QWLQ-CS and subscales ranged between 0.57 and 0.88 (Table 5). Subscales 2 ‘Perception of the work situation’ and 3 ‘Atmosphere in the work environment’ did not have an ICC_agreement_ ≥ 0.70. The level of agreement, which we assessed by SEM_agreement_, ranged between 9.59 and 13.89, except for subscale 5 ‘Problems due to the health situation’, which scored a higher SEM_agreement_ of 24.17. The mean differences of the overall QWLQ-CS score at baseline and follow-up did not statistically differ from zero (*p* = 0.694). The Bland and Altman plot (Fig. 1) displays the LoA with the means of baseline and four-week follow-up for the QWLQ-CS and the differences between these two measurements between the 95% confidence interval.

### Floor and ceiling effects

No cancer survivor had the lowest (0) or highest (100) possible overall QWLQ-CS score. The percentages of cancer survivors that scored the lowest or highest possible score on the subscales were <15%, so there were no floor or ceiling effects.

## Discussion

The items in the final version of the QWLQ-CS were reduced to 23 within a five-factor structure: 1) Meaning of work, 2) Perception of the work situation, 3) Atmosphere in the work environment, 4) Understanding and recognition in the organisation, and 5) Problems due to the health situation. The QWLQ-CS had adequate internal consistency, construct validity, and reproducibility at group level. No floor or ceiling effects were detected.

### QWLQ-Cs

In field study I we adequately performed EFA because the QWLQ-CS and its subscales had good internal consistency. In field study II we concluded that convergent validity was also good, although one hypothesis about correlations between a QWLQ-CS subscale and an existing subscale was not confirmed. The QWLQ-CS subscale 1 ‘Meaning of work’ had a low correlation with the COPSOQ subscale ‘Meaning of work’ (0.34). However, we followed the assumption that convergent validity is adequate when ≥75% of the hypotheses were confirmed [42], furthermore, the other correlations ranged between 0.53 and 0.70, which are moderate to strong correlations (*r* > 0.40) [29]. A possible explanation for the low correlation is that the COPSOQ subscale probably measured another construct than the QWLQ-CS subscale did. Although the items in both subscales looked similar, the latter subscale had included one different item: ‘Do you feel motivated and involved in your work?’. This might indicates a construct related to motivation and involvement in the organisation as well, whereas QWLQ-CS subscale 1 ‘Meaning of work’ does not. We recommend future research to study the convergent validity of this QWLQ-CS subscale with a different questionnaire that measures the same construct. For instance, the ‘Dedication’ subscale of the Utrecht Work Engagement Scale (UWES) [47].

Discriminative validity was assessed between cancer survivors and employed people without cancer or other physical/mental limitations affecting their job performance. There were statistically significant differences between the scores of the overall QWLQ-CS and subscale 5 ‘Problems due to the health situation’ for the two groups, with cancer survivors having a lower QWL score. This outcome is not surprising, as cancer survivors experience challenges in employment [8], which might influence QWL. However, cancer survivors do not always negatively differ from control groups. For instance, the quality of life of male cancer survivors (e.g. germ cell tumours) was similar to that of age-adjusted men [48]. Perhaps cancer survivors face more health issues at work (which might influence QWL) than in other areas of their lives that are influenced by quality of life. Furthermore, it seems that only health-related problems lower the QWL of cancer survivors. The scores of cancer survivors and employees without cancer did not differ on the subscales that contain generic items, only on the disease-specific items. Therefore, it might be interesting to study if the QWLQ-CS is also a valid QWL questionnaire for healthy employed people.

The reproducibility of the overall QWLQ-CS score was adequate with an ICC_agreement_ of 0.84, when ≥0.70 is acceptable for use at a group level [42]. However, subscale 2 ‘Perception of the work situation’ yielded a lower ICC_agreement_ of 0.57. Perhaps, this is caused by the homogeneity of the sample in regard to this subscale. By including cancer survivors with different backgrounds we assumed to have composed a heterogeneous sample. However, most cancer survivors had the same job for a long time, which might have influenced these items about self-efficacy, and made it difficult to distinguish between QWLQ-CS scores. Another parameter that determined the reproducibility of the QWLQ-CS was the SEM_agreement_, which ranged between 9.59 and13.89 (range 0–100) for the subscales. This is not uncommon, these SEM values are similar to quality of life outcomes on the SF-36 scale among COPD patients [49], and the SF-36 has been widely used because of its good psychometric properties. However, subscale 5 ‘Problems due to the health situation’ yielded a very high SEM_agreement_ of 24.17, which suggests that the repeated measures on this subscale for cancer survivors are far apart, and it is more difficult to achieve accuracy on the ‘true’ score and measure clinically important changes. A possible explanation for this high SEM is that the sample of cancer survivors differed in the experience of health-related problems, which may be a consequence of including cancer survivors who were diagnosed between 0 and 10 years ago. To test this assumption we should analyse the reproducibility of the QWLQ-CS only among cancer survivors who are diagnosed <1 year ago.

In sum, the reproducibility of the QWLQ-CS at group level is adequate. However, to use the QWLQ-CS at individual level, the reproducibility should be improved. For instance, by enhancing the true variance, which can be done by improving the scale design [28]. In regards to the QWLQ-CS, this would imply that the descriptions on the scale could be modified [28] or the number of response categories could be enhanced [50].

### Strengths and limitations

One strength of field study I is that we reduced the number of items in a systematic manner by performing an EFA. The steps taken in the EFA were statistically grounded, and decisions were made based on pre-set rules and extensive deliberation within the research group. By including cancer survivors with different demographic, health- and work-related backgrounds we developed a questionnaire with good psychometric properties that adequately measures QWL of different cancer survivors at group level.

Unfortunately, the results are less representative for cancer survivors with a different ethnic background, due to the lack of ethnic variety in the samples. Research in the USA revealed that racial or ethnic minorities of women with breast cancer were more likely to stop working compared to white women with breast cancer [51]. Perhaps this was also the reason for the lack of variety in ethnicity in our sample. However, the design of our study might also have led to non-participation: a systematic review on cancer survivorship research among minorities has shown that when working with minority populations, it is advisable to work inside the community and to draw on the help of respected leaders and involve minority members at every stage in the process [52]. This was not the case for our recruitment strategies, and therefore the results are not generalizable to cancer survivors with a different ethnic background. The implication for future research is that it is necessary to extend recruitment beyond hospitals and to recruit within the communities.

A methodological consideration concerns the number of factors in the QWLQ-CS. We used a combination of the scree plot and PA because previous literature suggested that there is no ‘golden standard’ [53]. The scree test provides a clear overview, but PA has proven to be more accurate in determining the number of underlying factors [53, 54]. Therefore, in case of any discrepancy, the final decision should be based on PA [54]. Ultimately, the two methods resulted in a unanimous number of factors: scree plot (*N* = 4) and PA (*N* = 8) with varying content and number of items within the factors. For instance, some factors in the PA contained a small number of items (*N* = 2), which is not desirable; a minimum of three items per factor is considered ideal [29]. Although no consistent evidence exists, it is suggested that PA might lead to overfactoring in case of smaller factor loadings and sample sizes [55]. Our sample size was not expected to be a problem as we had a ratio of 3.5 subjects per item, and a ratio of 4 subjects per item is recommended with a sample minimum of 100 [29]. However, QWL is a multi-dimensional construct and consists of items with very different contents which might lead to more low factor loadings. Eventually, we decided to determine the number of factors based on the outcomes of the scree test and PA, not only on the number of items, content and factor loadings. Therefore, we assume that the EFA did not result in an underfactoring or overfactoring of the number of factors in the QWLQ-CS. The adequate results of the psychometric properties of the QWLQ-CS support this conclusion.

### Implications for practice and research

The QWLQ-CS is ready for use in clinical and occupational healthcare and research settings at a group level. As the QWLQ-CS was developed in Dutch we do advise cross-cultural testing if used in other countries. Because the QWLQ-CS is a self-administered questionnaire, it is easy to use in practice and research. The QWLQ-CS can be used in studying differences in QWL between groups of cancer survivors. Previous research found that return to work of head and neck cancer survivors was associated with oral dysfunctions, and problems with social eating and social contacts [56]. It might be possible that different diagnoses lead to difficult problems at work that subsequently influence QWL. These results could be helpful in the development of tailored interventions aimed at return to work or work continuation of cancer survivors. In organisations with many employed cancer survivors, for instance, but also in research settings where rehabilitation interventions are evaluated at a group level and QWL may function as an additional research outcome. Furthermore, as some items such as ‘Because of my health situation I have problems in my work with fatigue and/or lack of energy’ seem to be relevant to other health problems besides cancer, it would be interesting to study if the QWLQ-CS can be applied to employees with other chronic diseases as well. Finally, it would be interesting in future research to measure the responsiveness of the QWLQ-CS evaluating whether it is possible to measure clinical changes in QWL which increases the applicability of the QWLQ-CS in practice.

## Conclusions

The five-factor QWLQ-CS with 23 items and adequate internal consistency, construct validity, and reproducibility at group level can be used in clinical and occupational healthcare and research settings.

## Additional files

Additional file 1: Psychometric properties. Psychometric properties of QWLQ-CS. (DOC 40 kb)

Additional file 2: English version of the Quality of Working Life Questionnaire for Cancer Survivors (QWLQ-CS). Word file of English version of the Quality of Working Life Questionnaire for Cancer Survivors (QWLQ-CS). (DOC 30 kb)

## Abbrevations

AMC: Academic Medical Center
CI: Confidence Interval
COPSOQ: Copenhagen Psychosocial Questionnaire subscales
COSMIN: hecklist COnsensus-based Standards for the selection of health Measurement INstruments
EFA: Exploratory Factor Analysis
EORTC: European Organisation for Research and Treatment of Cancer
ICC: Intraclass Correlation Coefficient
LoA: the Limits of Agreement
PA: Parallel Analysis
PCA: Principal Component Analysis
QWL: Quality of Working Life
QWLQ-CS: Quality of Working Life Questionnaire for Cancer Survivors
RTW-SE: return-to-work self-efficacy scale
SEM: Standard Error of Measurement
SF-36: the 36-Item Short Form Health Survey subscale
UWES: Utrecht Work Engagement Scale
VAS: Visual Analogue Scale
