# Supplementary material for: The quality of working life questionnaire for cancer survivors (QWLQ-CS): factorial structure, internal consistency, construct validity and reproducibility
Source: BMC Cancer. 2018 Jan 10;18:66. doi: 10.1186/s12885-017-3966-1 (PMC5763640; doi:10.1186/s12885-017-3966-1)
Supplement: Supplementary file 1 — Psychometric properties. Psychometric properties of QWLQ-CS. (DOC 40 kb) [file 12885_2017_3966_MOESM1_ESM.doc]

**Appendix A. Psychometric properties**

| **Measurement properties** | **Definition according to COSMIN** [57]* | **Parameters of measurement properties** | **Definition parameters according to COSMIN** [57]* | **I. Formula**  **II. Criteria for adequateness**  **III. Interpretability of outcome** |
| --- | --- | --- | --- | --- |
| Internal consistency | ‘The interrelatedness among the items; the extent to which items assess the same construct’ | Inter-item correlations | ‘Correlation between items within a factor’ | I. NA  II. 0.2-0.5  III. Correlation must not be too high: then items on the QWLQ-CS are too similar |
|  |  | Item-total correlations | ‘Indication of whether the items discriminate between cancer survivors on the construct’ | I. NA  II. >0.3  III. Low correlation means that the item does not discriminate between cancer survivors |
|  |  | Cronbach’s alpha | Coefficient of reliability | I. NA  II. 0.70-0.95  III. Higher Cronbach’s alpha indicates higher correlations between the items in the QWLQ-CS |
| Reproducibility: Test-retest reliability | ‘The degree to which cancer survivors can be distinguished from each other, despite measurement error’ | ICC_agreement_ | ‘Variance due to systematic differences between time points is included in random error variance’ | I. ơ^2^_cancer survivors_ / (ơ^2^_cancer survivors_ + ơ^2^_time_ + ơ^2^_residual_)  II. ≥ 0.70 (range between 0 and 1)  III. High reliability important for discriminative purposes if one wants to distinguish between cancer survivors |
| Reproducibility: Level of Agreement – measurement error | ‘How close the scores on repeated measures are, expressed in the unit of the measurement scale at issue’ | Standard Error of Measurement (SEM) | ‘Measure of how far apart the outcomes of the repeated measurements are; it is the SD around a single measurement’ | I. SEM_agreement_ = √(ơ^2^_time_ + ơ^2^_residual_)  II. Scale 0-100  III. See LoA |
|  |  | Limits of agreement (LoA) | ‘Indication of the size (magnitude) of measurement error’ | I. LoA = ­(mean_change_) ± 1.96 x SD_change_)  II. If 95% of the differences between repeated measurements fall between limits of agreement: due to measurement error. Changes that fall outside the limits of agreement: possible real change  III. Small measurement error is required for evaluative purposes in which one wants to distinguish clinically important changes from measurement error. |
| Construct validity: Convergent validity | ‘The degree to which the construct that is measured in the QWLQ-CS is related to similar constructs’ | A priori hypotheses | Hypotheses about the relationship of scores on the QWLQ-CS with the scores on other instruments measuring similar constructs (correlations). | I. NA  II. ≥ 75% of hypotheses are confirmed  III. If hypothesis are confirmed; the QWLQ-CS is a valid questionnaire for cancer survivors. |
| Construct validity: Discriminative validity | ‘The ability of the QWLQ-CS to discriminate between groups that are expected to differ in outcomes scores’ | A priori hypotheses | Hypotheses about statistical differences in the QWLQ-CS scores between subgroups | I. NA  II. ≥ 75% of hypotheses are confirmed  III. If hypothesis are confirmed; the QWLQ-CS is a valid questionnaire for cancer survivors. |

^*^  Mokkink LB, Terwee CB, Patrick DL, Alonso J, Stratford PW, Knol DL, et al. The COSMIN study reached international consensus on taxonomy, terminology, and definitions of measurement properties for health-related patient-reported outcomes. J Clin Epidemiol. 2010;63:737-45.
